# The outcomes of ultrafiltration in on-pump versus off-pump coronary artery bypass grafting in patients with renal impairment

**DOI:** 10.1186/s13019-022-01976-7

**Published:** 2022-08-31

**Authors:** Amarit Phothikun, Weerachai Nawarawong, Apichat Tantraworasin, Thitipong Tepsuwan

**Affiliations:** 1 Cardiovascular and Thoracic Surgery Unit, Department of Surgery, Faculty of Medicine, Chiang Mai University, Chiang Mai, Thailand; 2 General Thoracic Surgery Unit, Department of Surgery, Faculty of Medicine, Chiang Mai University, Chiang Mai, Thailand; 3 Clinical Epidemiology and Clinical Statistic Center, Faculty of Medicine, Chiang Mai University, Chiang Mai, Thailand; 4 Clinical Surgical Research Center, Chiang Mai University, Chiang Mai, Thailand

**Keywords:** Cardiopulmonary bypass, Ultrafiltration, CABG, OPCAB

## Abstract

**Objective:**

In chronic kidney disease (CKD), using cardiopulmonary bypass (CPB) may contribute to renal dysfunction. Off-pump coronary artery bypass grafting (OPCAB) is one technique that preserved renal function, but the procedure may not be possible in certain situations. The ultrafiltration (UF) can remove excess fluid and inflammatory mediators that result from exposure to the CPB. Coronary artery bypass grafting (CABG) with UF could be an alternative way to preserve renal function.

**Method:**

A retrospective study of CKD patients who underwent CABG. The renal outcomes were compared between the patients who underwent CABG with UF and OPCAB. A repeated measure adjusted by propensity score was used for comparing the renal outcome. Univariable and multivariable logistic regression was used to identify the risk factors for acute renal failure (AKI) and adverse outcomes.

**Results:**

From January 2009 and June 2020, there were 220 CKD patients, 109 (49.55%) patients underwent CABG with UF, and 111 (50.45%) patients underwent OPCAB. There were statistically significant differences in the change of the average level of creatinine between CABG with UF (increased + 0.09 mg/dl) and OPCAB (decreased − 0.05 mg/dl) (*p* = 0.043). Also, patients who underwent CABG with UF had a significantly increased risk for AKI (OR 5.38, 95%CI 1.09, 26.5).

**Conclusion:**

The UF adjunct technique in CABG with CPB tends to provide a lower protective effect for renal function and had a significantly higher incidence of post-cardiac surgery AKI when compared to OPCAB. If technically feasible, OPCAB would be a preferable choice for CKD patients.

*Study registration number*: SUR-2562-06607/Research ID: 6607.

## Introduction

In CKD patients, several studies reveal that patients who underwent cardiac surgery with CPB had a higher incidence of acute kidney injury during the post-operative period [1]. Using CPB may contribute to renal dysfunction resulting from the consequences of significant hemodilution and post-bypass systemic inflammatory response syndrome (SIRS) [2].

One strategy to avoid adverse effects from the CPB is to use the non-CPB CABG technique or Off-pump CABG (OPCAB). Some evidence showed that OPCAB can provide better renal protection and reduce incidences of post-operative AKI [3, 4]. However, many cardiac surgeons prefer to perform CABG with CPB over OPCAB. OPCAB still had unclear benefits of associated mortally and long-term outcomes when compared to conventional CABG [5]. Moreover, OPCAB is a technically demanding procedure that may not be possible in certain situations such as severe hemodynamic instability or those who require concomitant procedures such as ischemic mitral valve surgery or surgical ventricular restoration.

Another strategy has been developed to protect the kidney while inevitably running the CPB circuit. Ultrafiltration is a technique that uses a semipermeable membrane. These membranes are connected in a CPB circuit to remove excess fluid and create a hemoconcentration [2]. It works similarly to hemodialysis, except it cannot filtrate waste products from serum. Moreover, ultrafiltration can reduce inflammatory mediators, therefore ultrafiltration could reduce SIRS [6].

Several studies found that patients who used ultrafiltration during CPB had a better post-operative outcome than patients who did not use it. They also had higher urine volume, a lower amount of blood transfusion, and lower adverse outcomes in post-operative periods [1, 7–12]. Although there are currently strong evidence to suggest that ultrafiltration during the period of CPB might have a main impact on the postoperative renal function, the use of ultrafiltration may have greater benefits when added to CPB than CPB alone, especially in CKD patients.

Because there were CABG with ultrafiltration strategy that used for preserved renal function in unsuitable for OPCAB patients (or in none-OPCAB preferred surgeons). Therefore, this study was designed to compare the post-operative renal function in CKD patients between those who underwent CABG with ultrafiltration and those who underwent OPCAB. However, the question remains which techniques between OPCAB and CPB with UF would be a preferable choice for CKD patients who undergoing CABG to prevent deterioration of renal function. We present the following article in accordance with the TREND reporting checklist.

## Materials and methods

### Patients

This therapeutic research was conducted with a retrospective observational cohort design in Maharaj Nakorn Chiang Mai Hospital, Faculty of Medicine, Chiang Mai University, Thailand, and was approved by the Research Ethics Committee Faculty of Medicine, Chiang Mai University. The target population was coronary artery disease patients who underwent CABG including all techniques (On-CPB & off-CPB) from January 2009-June 2020. The inclusion criteria were patients older than 18 years old with underlying CKD stages IIIa-b, IV, and V (Form Kidney Disease Improving Global Outcome (KDIGO) stage of CKD) [13, 14]. Patients whose operative technique were converted intraoperatively from OPCAB to on-pump CABG were excluded from this study.

Six cardiac surgeons were performing CABG in this study and all surgeons perform both OPCAB and On-CPB CABG techniques. In each surgeon, there was a difference between the volume of each type of technique. Therefore, the operated surgeon variable was used as a confounding factor in the statistical calculation. Patients’ selection was based on the surgeon’s preference, ultrafiltration was always included in every patient who was decided to underwent On-CPB CABG. A total of 220 cases were enrolled in this study and divided into two groups; 111 (50.45%) patients underwent OPCAB, and 109 (49.55%) patients underwent CABG with UF.

Due to the retrospective study, there were differences in baseline demographic data between the 2 groups of patients. If any demographic variables have been defined as confounding factors, those variables were used for calculating the propensity score (detail in statistical analysis).

### Operative technique

#### OPCAB group

After median-sternotomy and conduit harvested, Heparin 1.5 mg/kg was administrated to the patients to keep ACT at more than 300 s. The bypass grafting was performed and each graft was evaluated for the quality of anastomosis by transit time flow measurement.

#### UF group

For the perfusion technique, CPB was performed with a Stockert Roller pump (Stockert Instrument Gmb H) and Inspire8 (LivaNova, Germany) oxygenator. The circuit was primed with Acetate Ringer’s solution and 20% Mannitol. Patients were heparinized with intravenous heparin with an initial dose of 3 mg/kg and adjusted to maintain an activated clotting time > 400 s. While CPB was running, the blood flow was maintained from 2.4 to 3.0 L/min/m^2^ and used a normothermic state (around 35 °C by rectal temperature). Target hematocrit under CPB was maintained between 20 and 25%. Myocardial protection strategy was performed with Buckberg blood cardioplegia with an initial dose of a total volume of 1800 ml and repeated with 550 ml every 20 min.

For conventional ultrafiltration (CUF), the inlet of the CUF circuit was connected between the arterial line bridge and the outlet tubing with a 3-way connector to the venous reservoir. An AQUAMAX^®^ HF19 polyethersulfone membrane was used as a filter through where blood flow was maintained by the rate of the main CPB circuit’s flow rate. CUF was always performed while CPB was running and stopped immediately before CPB weaning.

After termination of CPB, Modified ultrafiltration (MUF) was performed by using the same filter as the CUF circuit. Ascending aortic cannula was used as an efferent line that allowed blood to pass at a rate of 150–300 mL/min controlled by a roller pump. This blood flowed through the filtered membrane and was sent back into circulation via a venous cannula into the right atrium continuously for 10 min. The MUF technique can filtrate a larger amount of excess water and more concentrated blood than the CUF [1, 2]. Aortic and venous cannula was then removed after MUF was completed.

### Endpoint

BUN, Cr, and Crcl/eGFR were the most practical and useful laboratory values used to evaluate renal function in this study [14–16]. When the patients were admitted to the hospital, blood samples were taken to evaluate pre-op renal function (Baseline). After finishing the operation, the patients were transferred to the Cardiac Intensive Care Unit (ICU). Renal function was evaluated again when patients arrived (Immediate post-operation) and 24-h post-operation (Day 1). Finally, after patients were discharged from the hospital, the renal function was evaluated again at 30 days post-operation during a follow-up appointment in the outpatient department (Day 30).

The severity of AKI was also used to evaluate the renal function referencing KDIGO criteria. AKI means an increase in serum Cr more than 1.5 times compare to the baseline which presumes parameters within 7 days after monitoring and within 3 months before transform to CKD [14]. The AKI stage by KDIGO criteria was used to evaluate the severity of post-operation kidney injury at 24 h (Day1) and 30 days (Day 30).

### Statistical analysis

Statistical analyses were performed on software STATA version 16.1. The sample size was calculated by test comparing two independent means based on the studies that the design and the outcome were resemble this study [6]. Categorical data were described as frequency and percentages and chi-square test was applied for group comparisons. Continuous data presented with mean and standard deviation (SD). Paired Student’s t-test was applied for comparisons of variables between the groups.

Logistic regression was used to calculate a propensity score (PS), which evaluates confounding by indication. The variables included in the model for PS were Canadian Cardiovascular Society Classification, EuroSCORE II, Use of Angiotensin-converting enzyme inhibitor drugs/Angiotensin receptor blockers drugs, Pre-operative Ejection fraction, Dyslipidemia, Chronic kidney disease stage, coronary artery disease, pre-op Intra-aortic balloon pump, type of surgery and operated surgeons; the score was then divided into quintiles, called PS-groups. A comparison of the differences in continuous data variables (renal function) between the groups was calculated using Repeated Measure Mixed Model stratified by PS-group and adjusted by other confounding factors. And the differences were presented with “Change per follow-up”. Ordered logistic regression and binary risk regression analysis were used to identify the risk factor for increased severity of AKI and other postoperative outcomes. The selection of variables for multivariate analysis was based on clinical relevance or significance in univariate analysis. All statistical differences were considered significant at *p* < 0.05.

## Result

### Demographic and operative data

The demographic data of patients between groups were shown in Table 1. UF group had a significantly more advanced stage of CKD, higher baseline creatinine, and lower eGFR. UF group also had higher predicted mortality by EuroSCORE II.

**Table 1:** Patient demographic & operative data

	UF *n* = 109	OPCAB *n* = 111	*p* - Value
Age (year, mean ± SD)	64.23 ± 8.85	66.62 ± 9.41	0.054
Male, *n* (%)	83 (76.15)	70 (63.06)	0.041
NYHA functional class, *n* (%)			0.075
Class I	9 (8.26)	18 (16.22)	
Class II	73 (66.97)	57 (51.35)	
Class III	24 (22.02)	29 (26.13)	
Class IV	3 (2.75)	7 (6.31)	
CCS class, *n* (%)			< 0.001
Class 0	21 (19.27)	8 (7.21)	
Class I	48 (44.04)	33 (29.73)	
Class II	30 (27.52)	40 (36.04)	
Class III	7 (6.42)	21 (18.92)	
Class IV	3 (2.75)	9 (8.11)	
Body weight (kg, mean ± SD)	59.8 ± 10.32	60.0 ± 13.37	0.918
Body height (cm, mean ± SD)	159.9 ± 7.89	156.4 ± 17.49	0.059
EuroSCORE II (%, mean ± SD)	4.86 ± 3.82	3.72 ± 4.67	0.048
Pre-op creatinine (mg/dL, Mean ± SD)	4.22 ± 3.02	2.25 ± 2.14	< 0.001
Pre-op eGFR (ml/min, Mean ± SD)	22.70 ± 16.44	40.59 ± 15.72	< 0.001
Ejection fraction (%, Mean ± SD)	48.1 ± 15.55	53.0 ± 13.4	0.012
Previous medication, *n* (%)			
Betablocker	76 (69.72)	64 (57.66)	0.070
ACEI/ARBs	28 (25.69)	64 (57.66)	< 0.001
Calcium channel blocker	42 (38.53)	40 (36.04)	0.781
Nitrate	61 (55.96)	84 (75.68)	< 0.001
Statin	94 (86.24)	101 (90.99)	0.294
Underlying disease, *n* (%)			
Diabetes mellitus with no Insulin therapy	25 (22.93)	35 (31.53)	0.521
Insulin injection therapy	28 (25.68)	29 (26.13)	0.532
Hypertension	105 (96.33)	102 (91.89)	0.252
Dyslipidemia	95 (87.15)	83 (74.77)	0.025
Old cerebrovascular disease	6 (5.5)	9 (8.11)	0.594
History of myocardial ischemia	23 (21.1)	17 (15.32)	0.297
COPD	1 (0.9)	2 (1.8)	0.507
Chronic kidney disease, *n* (%)			< 0.001
Stage III (IIIa & IIIb)	34 (31.19)	83 (74.77)	
Stage IV	25 (22.93)	15 (13.51)	
End stage renal disease	49 (44.95)	13 (11.71)	
Coronary artery disease, *n* (%)			0.066
Single vessel	5 (4.58)	1 (0.9)	
Double vessel	19 (17.43)	11 (9.91)	
Triple vessel	85 (79.43)	99 (89.19)	
Left main disease, *n* (%)	36 (33.02)	40 (36.04)	0.372
Pre-op IABP, *n* (%)	4 (4.6)	17 (15.32)	0.005
Operated surgeon, *n* (%)			< 0.001
1	9 (8.26)	73 (65.77)	
2	53 (48.62)	6 (5.41)	
3	16 (14.68)	26 (23.42)	
4	2 (1.83)	1 (0.9)	
5	13 (11.93)	5 (4.5)	
6	16 (14.68)	0 (0)	
Status of surgery, *n* (%)			0.939
Elective	98 (89.90)	98 (88.29)	
Urgent	8 (7.33)	10 (9.01)	
Emergency	3 (2.75)	3 (2.7)	
Preoperative MAP (mmHg, Mean ± SD)	84.54 ± 12.97	89.91 ± 12.49	0.002
Number of anastomosis (Mean ± SD)	4.15 ± 1.21	4.06 ± 1.14	0.559
Use of radial arterial graft, *n* (%)	47 (43.11)	56 (50.45)	0.284
Operative time (minutes, Mean ± SD)	298.56 ± 85.86	260.52 ± 83.4	0.001
Total hemofiltration time (min, Mean ± SD)	9.62 ± 0.95	-	-
Ultrafiltration volume (ml, Mean ± SD)	1328.35 ± 776.97	-	-
Peri-mediastinal drainage in first 24 h. (ml, Mean ± SD)	402.67 ± 300.5	475.4 ± 211.6	0.295
Blood transfusion, *n* (%)	45 (41.28)	46 (41.4)	0.780
Number of post-op PRC usage (unit, mean ± SD)	1.60 ± 1.04	1.39 ± 0.6	0.512
Early re-operation, *n* (%)	6 (5.55)	1 (0.9)	0.064
Post-op inotropic drug			
Norepinephrine	49 (44.95)	38 (34.2)	0.068
Adrenaline	23 (21.1)	6 (5.41)	< 0.001
Dobutamine	13 (11.92)	10 (9.0)	0.516
Milrinone	21 (19.26)	4 (3.6)	< 0.001
Propensity scores (mean ± SD)	0.75 ± 0.25	0.25 ± 0.25	< 0.001

*OPCAB*, Off-pump coronary artery bypass; *UF*, ultrafiltration; *BUN*, Blood urea-nitrogen; *NYHA*, New York Heart Association functional classification; *CCS*, Canadian Cardiovascular Society; *EuroSCORE II*, the European system of Cardiac Operative Risk Evaluation II; *Pre-op*, Pre-operative; *ACEI/ARBs*, Angiotensin converting enzyme inhibitor drugs/Angiotensin receptor blockers drugs; *COPD*, Chronic obstructive pulmonary disease; *IABP*, Intra-aortic balloon pump, *MAP*, Mean arterial pressure; *PRC*, Packed red cell

Statistically significant at *p* < 0.05

### Renal function

**Fig. 1:**
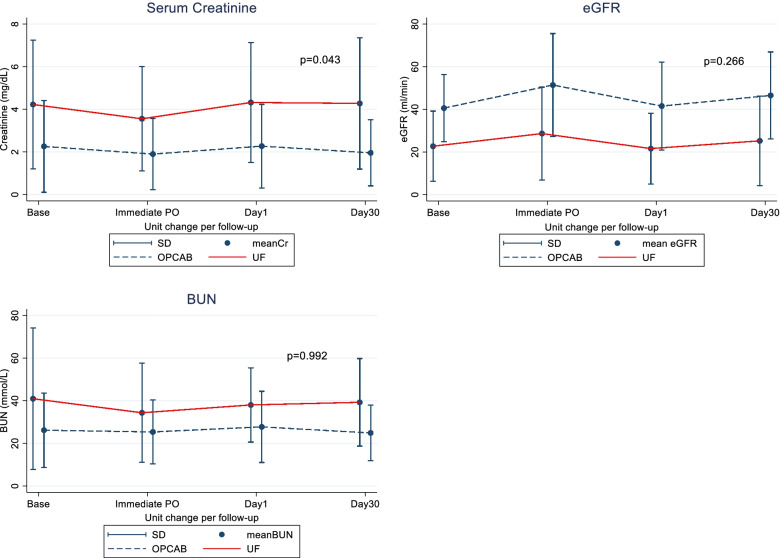
Linear Graft for the change of renal function per follow-up among the 2 groups. UF, ultrafiltration; OPCAB, Off-pump coronary artery bypass; Cr, creatinine; eGFR, estimated glomerular filtration rate; BUN, Blood urea-nitrogen; SD, standard deviation; Base, baseline pre-operative; Immediate PO, Immediate post-operative. *p* < 0.05: statistically significant different

From Fig. 1, both groups had Cr level lower than baseline creatinine immediately after operations (UF 3.6 mg/dL, OPCAB 1.9 mg/dL) and the levels returned to baseline after post-op Day 1(UF 4.3 mg/dL, OPCAB 2.3 mg/dL). On post-op day 30, the Cr level in the UF group remained stable while the level in the OPCAB group decrease significantly lower than baseline (UF 4.3 mg/dL, OPCAB 1.9 mg/dL). The difference in renal function between the two study groups was compared by the change of value during follow-up which was represented by unit change per follow-up (Table 2). There was a significant difference in the change in Cr level between the UF and OPCAB groups (*p* = 0.043). Which was interpreted as an increase of Cr level by 0.44 mg/dL throughout the follow-up period in the UF group and a decrease in Cr level by 0.05 mg/dL throughout the follow-up period in the OPCAB group. However, these changes were not significant differences between the two groups regarding eGFR and BUN level.

**Table 2:** Post-operative renal function analyzed by repeated measure mixed model stratified by propensity score

Variables	UF			OPCAB			*p*-value of change between groups
	Changed per follow up	*p*	95% CI	Changed per follow up	*p*	95% CI	
*All patients*							
Creatinine	+ 0.09	0.071	− 0.01, + 0.19	− 0.05	0.293	− 0.15, + 0.04	0.043
eGFR	+ 0.05	0.915	− 0.88, + 0.98	+ 0.79	0.092	− 0.12, + 1.71	0.266
BUN	− 0.13	0.854	− 1.58, + 1.31	− 0.14	0.842	− 1.58, + 1.29	0.992
*ESRD subgroup analysis*							
Creatinine	+ 0.15	0.204	− 0.08, + 0.38	− 0.26	0.338	− 0.8, + 0.27	0.167
eGFR	− 0.33	0.158	− 0.79, + 1.3	+ 0.30	0.578	− 0.76, + 1.37	0.286
BUN	− 0.18	0.89	− 2.8, + 2.43	− 5.16	0.098	− 11.2, + 0.95	0.142

Creatinine (mg/dL), *eGFR*, Estimated Glomerular filtration rate(ml/min), *BUN*: Blood urea-nitrogen (mmol/L), *OPCAB*: Off-pump coronary artery bypass, *UF*, ultrafiltration

Statistically significant at *p* < 0.05

**Fig. 2:**
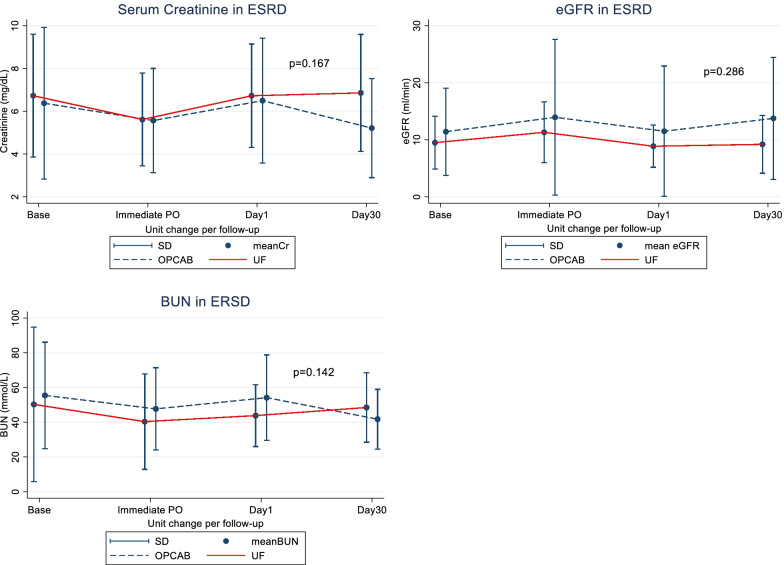
Linear Graft for the change of renal function per follow-up among the 2 ESRD sub-groups. ESRD, end-stage renal disease; UF, ultrafiltration; OPCAB, Off-pump coronary artery bypass; Cr, creatinine; eGFR, estimated glomerular filtration rate; BUN, Blood urea-nitrogen; SD, standard deviation; Base, baseline pre-operative; Immediate PO, Immediate post-operative. *p* < 0.05: statistically significant different

ESRD subgroup analyses were used to compare the overt renal function. A new propensity score was calculated from separate data for calculating the final renal function result. In the UF with ESRD subgroup (Table 2 and Fig. 2), there was no significant difference in the change of Cr level between the UF and OPCAB group (*p* = 0.167). Which was interpreted as an increase of Cr level by 0.15 mg/dL throughout the follow-up period in the UF group and a decrease in Cr level by 0.26 mg/dL throughout the follow-up period in the OPCAB group. And these changes were not significant differences between the two groups regarding the eGFR and BUN levels.

### Acute kidney injury

On immediate post-operative and post-operative Day 1, the UF group had a higher incidence of AKI (Immediate: UF 4.2%, OPCAB 1.8% *p* = 0.117, post-op Day 1: UF 11%, OPCAB 4.5% *p* = 0.169), but there was not a statistically significant difference. On post-operative Day 30, the UF group had increased the incidence of AKI in a statistically significant way compared to OPCAB (Day 30: UF 11.9%, OPCAB 2.7% *p* = 0.014).

Multivariable risk factor analysis for an increased stage of AKI was present in Table 3. The important result was on post-operative Day 30, patients who underwent CABG with UF had an increased chance of AKI KDIGO stage 5.38 times compared to OPCAB with a statistically significant difference (*p* = 0.038). The other risk factors to increase the AKI KDIGO stage were not statistically significant.

**Table 3:** Risk factor of post-operative KDIGO acute kidney injury stage changing of CABG with ultrafiltration versus OPCAB by univariable and multivariable ordered logistic regression

	Univariable			Multivariable		
	OR	*p*	95%CI	OR	*p*	95%CI
*Post-op day 1*						
UF	2.64	0.077	0.89–7.77	2.51	0.180	0.65–9.68
*Post-op day 30*						
UF	4.9	0.015	1.35–17.7	5.38	0.038	1.09–26.53
Age > 59	0.5	0.196	0.17–1.43			
Male	0.97	0.963	0.32–2.92			
CCS class > 1	2.4	0.406	0.30–18.8			
Euro II score > 4	2.3	0.112	0.82–6.42			
Pre-op ACEI/ARBs	0.29	0.063	0.08–1.07			
Pre-op Nitrate	1.61	0.419	0.50–5.20			
DLP	0.69	0.552	0.21–2.28			
CKD	2.43	0.005	1.31–4.52	1.49	0.252	0.75–2.99
CKD stage3	0.26	0.024	0.08–0.83	0.50	0.312	0.13–1.90
CKD stage4	0.62	0.537	0.13–2.83			
ESRD	4.96	0.003	1.72–14.3	1.98	0.312	0.52–7.45
Pre-op IABP	2.03	0.992	0.05–0.14			

*OR*, Odd ratio; *Post-op*, post-operative; *UF*, ultrafiltration; *CCS*, Canadian Cardiovascular Society grading of angina pectoris; *Pre-op*, pre-operative; *ACEI/ARBs*, Angiotensin-converting enzyme inhibitors/angiotensin II receptor blockers drug; *DLP*, dyslipidemia; *CKD*, Chronic kidney disease; *ESRD*, end stage renal disease; *IABP*, Intra-aortic balloon pump; *CABG*, coronary artery bypass grafting

Statistically significant at *p* < 0.05

### Post-operative clinical outcomes

Multivariable risk factor analysis for post-operative outcomes shows that the patients who underwent CABG with UF were 2.46 times more likely to develop post-op arrhythmia (*p* < 0.001) compared with patients who underwent OPCAB. And Post-op inotropic drugs were used higher in the UF group (*p* = 0.020) (Table 4).

**Table 4:** Risk factor of post-operative clinical outcome of CABG with ultrafiltration versus OPCAB by univariable and multivariable risk regression

	Univariable			Multivariable		
	RR	*p*	95%CI	RR	*p*	95%CI
New hemodialysis	1.92	0.345	0.49–7.49			
Post-op arrhythmia	2.99	< 0.001	2.12–4.22	2.46	< 0.001	1.59–3.80
Post-op inotropic drugs	1.41	0.002	1.13–1.76	1.44	0.020	1.05–1.97
CVA	2.04	0.559	0.18–22.1			
Sepsis	3.39	0.058	0.96–12.0			
Ventilator time > 24 h	4.24	0.060	0.93–19.15			
ICU > 48 h	2.13	0.079	0.92–4.97			
Hospital > 14 days	1.27	0.156	0.91–1.77			
30 days mortality	2.04	0.405	0.38–10.89			

*RR*, Risk ratio; *Post-op*, post-operative; *LOCS*, Low cardiac output syndromes; *CVA*, Cerebrovascular accident or stroke; *ICU*, Intensive care unit

Statistically significant at *p* < 0.05

## Discussion

Chronic renal failure increases the mortality and morbidity in patients undergoing cardiac surgery [17]. For CABG, there are many strategies used to reduce and protect against renal impairment during surgery. OPCAB is one technique that has renal protective effects and can reduce post-operative AKI compared to conventional CABG [3, 4]. Although OPCAB seems to be the perfect technique for avoiding renal dysfunction, OPCAB is a technically demanding procedure that may not be possible in certain situations. In some situations, the operation needs to change from OPCAB to conventional CABG due to the patient's hemodynamics being unstable.

CABG with UF technique is another adjunctive protective strategy. The main advantage of UF was that it removed excess fluid and inflammatory mediators, that were created during the CPB period [9–12]. For CKD patients who need to do CABG, the UF adjunct technique might have a greater benefit than using conventional CABG alone.

The major findings of this comparative study were differences in baseline renal function between the UF and the OPCAB groups. Demographic data shows the patients in the UF group had a higher stage of CKD. Mean Cr and BUN levels were higher and the mean of eGFR was lower in the UF group, thus representing inherited selection bias from the retrospective study. So, the difference in baseline renal function was adjusted by statistical method before comparing between the two groups. The propensity score and repeated measure were used to adjust the mean difference to make it equal in the statistic method.

During the immediate post-operative period, all renal functions in both groups had a better result when compared with the pre-operative baseline. The change in renal function at the immediate post-operative time especially creatinine level was probably caused due by hemodilution. It is essential to maintain adequate preload in OPCAB by volume administration to prevent hemodynamic deterioration during heart manipulation and elevation. While in the UF group, even ultrafiltration reduced the excess fluid from CPB, there was still an excessive volume that remained in the body. These hemodilution effects have been reported by some prospective randomized studies of post-operative renal function between ultrafiltration and non-ultrafiltration in CABG patients found all patients which showed that serum creatinine decreased immediately after surgery in both conventional and ultrafiltration groups [7].

On post-operative Day 1, all renal function values trended to be closer to the pre-operative baseline, because the hemodilution effect was resolved due to post-operative management. Both the UF and OPCAB groups had no difference in renal function at early postoperative period but differences were detected on post-operative Day 30. In the UF group, the renal function was equal to the pre-operative baseline. But in the OPCAB group, the renal function was better than the baseline; Cr and BUN were lower and eGFR was higher than the pre-operative value.

The overall change in Cr level differed significantly between groups: an increase in the UF group and a decrease in the OPCAB group. Although the change was small, this might indicate that the effect of intraoperative UF could not reduce the overall renal dysfunction at one month. Even though it can reduce the excess fluid, the adverse effects of inflammatory response from CPB exist not only in the immediate early post-operative period but also persists later in the follow-up period. Tang et al. showed in a study with case-controlled trials between OPCAB and conventional CABG, that serum creatinine was significantly lower and creatinine clearance was higher in the OPCAB patients. The results from this trial show the increase in Cr level was correlated with the use of CPB [18]. Another study from Popatov et al. a randomized trial, also reported that creatinine clearance was significantly higher in the no-CPB group than in the CPB group from intraoperative until 48 h post-operatively. Moreover, this study used urinary n-acetyl-b-glucosaminidase (NAG), a renal tubular damage marker, that is significantly lower in OPCAB [19].

Multivariable analysis shows if the patient underwent CABG with UF, there was a significantly higher risk of post-operative AKI compared with OPCAB. This result is similar to previous studies in that OPCAB had 40 percent lower odds for post-operative AKI than CABG [3, 20]. Some studies even found that exposure to UF increases the risk of AKI, despite similar death stroke and bleeding [2, 21]. Moreover, multivariable ordinary logistic regression analysis shows the UF was a factor that increased the stage of AKI (by KDIGO) significantly higher than OPCAB, especially at post-operative Day 30. This further confirmed the persistent continuous adverse effects of CPB on renal function.

For other post-operative results, the UF group had significantly increased the risk for post-operative AF, inotropic usage, and longer ventilator time. These also could be explained by the effects of systemic inflammatory response that happened during CPB causing susceptibility to cardiac arrhythmias, lower peripheral vascular resistance, vasodilatation, and pulmonary injury. Although UF theoretically removed these inflammatory mediators and alleviate their adverse reaction, it is not known how much UF can reduce and how many inflammatory mediators remain in the circulation [22]. The remaining inflammatory mediator is still active and causes the body’s response.

Although there was small clinical significance without clear clinical benefit in terms of renal function, OPCAB might still be the preferable choice in CKD patients because it can also prevent other complications from CPB and SIRS such as atrial fibrillation and vasoplegic syndrome. This might imply the advantages of OPCAB techniques beyond renal protection in patients with CKD.

For the ERSD subgroup, there was no significant difference in postoperative change of Cr level between groups. Because this group of patients requires regular dialysis, the reno-protective effect of OPCAB was diminished. However, post-cardiac arrhythmia was lower in the OPCAB group. Whether or not OPCAB would reduce morbidities and mortality in this specific subset of patients. Many studies reveal that OPCAB has a greater benefit to ESRD patients in mortality and morbidities than on-CPB patients [3, 5, 16, 17, 23, 24].

Finally, there are several limitations of this study. The main limitation was that this study was conducted retrospectively and therefore there may be many heterogeneities between the study groups. As seen in the demographic data, in the UF group all patients tended to have a higher CKD stage when compared with the OPCAB group and the change in the renal function at each CKD stage was different. And baseline of pre-operative was different from different stages of CKD. However, we used statistical methods to correct the confounding factor and used the statistical way to adjust an unequal baseline to be proper to compare. Another limitation would be that the follow-up period for assessing renal function might be too short. When renal dysfunction occurs, it needs time to resolve back to the baseline or turn into a new higher baseline. Therefore, 30 days might not be enough to assess the final adjustment of renal function.

## Conclusion

Although using a protective strategy in CABG with CPB by ultrafiltration, renal function using the OPCAB technique is still better, especially for the change of serum creatinine level. The UF adjunct technique significantly increases the creatinine level when compared to the OPCAB technique which decreases the creatinine level during the post-operative follow-up period. The reno-protective effects of OPCAB indicate the adverse inflammatory effects from CPB that could lead to other complications affecting the overall outcome. Therefore, OPCAB technique may be suitable for patients with impaired kidney function. However, further studies with randomized control trials or larger sample sizes are warranted to support the results of this study.

## Data Availability

The datasets used and/or analyzed during the current study are available from the corresponding author on reasonable request.

## Abbreviations

CABG: Coronary artery bypass grafting
OPCAB: Off-pump coronary artery bypass
CPB: Cardiopulmonary bypass, pump
UF: Ultrafiltration
BUN: Blood urea nitrogen
Cr: Creatinine
eGFR/Crcl: Estimated glomerular filtration rate or creatinine clearance
CKD: Chronic kidney disease
AKI: Acute kidney injury
